# Different IMRT solutions vs. 3D-Conformal Radiotherapy in early stage Hodgkin’s lymphoma: dosimetric comparison and clinical considerations

**DOI:** 10.1186/1748-717X-7-186

**Published:** 2012-11-02

**Authors:** Christian Fiandra, Andrea Riccardo Filippi, Paola Catuzzo, Angela Botticella, Patrizia Ciammella, Pierfrancesco Franco, Valeria Casanova Borca, Riccardo Ragona, Santi Tofani, Umberto Ricardi

**Affiliations:** 1 University of Turin, Department of Oncology, Radiation Oncology Unit, Turin, Italy; 2 Radiation Oncology Department, Tomotherapy Unit, Ospedale Regionale ‘U. Parini’, AUSL Valle d’Aosta, Aosta, Italy; 3 Medical Physics Unit, Ospedale Regionale U. Parini, Aosta, Italy

## Abstract

**Background:**

Radiotherapy in Hodgkin’s Lymphoma (HL) is currently evolving with new attempts to further reduce radiation volumes to the involved-node concept (Involved Nodes Radiation Therapy, INRT) and with the use of intensity modulated radiotherapy (IMRT). Currently, IMRT can be planned and delivered with several techniques, and its role is not completely clear. We designed a planning study on a typical dataset drawn from clinical routine with the aim of comparing different IMRT solutions in terms of plan quality and treatment delivery efficiency.

**Methods:**

A total of 10 young female patients affected with early stage mediastinal HL and treated with 30 Gy INRT after ABVD-based chemotherapy were selected from our database. Five different treatment techniques were compared: 3D-CRT, VMAT (single arc), B-VMAT (“butterfly”, multiple arcs), Helical Tomotherapy (HT) and Tomodirect (TD). Beam energy was 6 MV, and all IMRT planning solutions were optimized by inverse planning with specific dose-volume constraints on OAR (breasts, lungs, thyroid gland, coronary ostia, heart). Dose-Volume Histograms (DVHs) and Conformity Number (CN) were calculated and then compared, both for target and OAR by a statistical analysis (Wilcoxon’s Test).

**Results:**

PTV coverage was reached for all plans (V_95%_ ≥ 95%); highest mean CN were obtained with HT (0.77) and VMAT (0.76). B-VMAT showed intermediate CN mean values (0.67), while the lowest CN were obtained with TD (0.30) and 3D-CRT techniques (0.30). A trend of inverse correlation between higher CN and larger healthy tissues volumes receiving low radiation doses was shown for lungs and breasts. For thyroid gland and heart/coronary ostia, HT, VMAT and B-VMAT techniques allowed a better sparing in terms of both D_mean_ and volumes receiving intermediate-high doses compared to 3D-CRT and TD.

**Conclusions:**

IMRT techniques showed superior target coverage and OAR sparing, with, as an expected consequence, larger volumes of healthy tissues (lungs, breasts) receiving low doses. Among the different IMRT techniques, HT and VMAT showed higher levels of conformation; B-VMAT and HT emerged as the planning solutions able to achieve the most balanced compromise between higher conformation around the target and smaller volumes of OAR exposed to lower doses (typical of 3D-CRT).

## Background

The overall prognosis of patients affected with early stage Hodgkin’s lymphoma (HL) is excellent, with an overall 15-year survival rate of more than 80%. A combined modality treatment approach, consisting of a brief chemotherapy (2 to 4 cycles of Adriamycin, Bleomycin, Vinblastine, Dacarbazine: ABVD cycle) followed by 20–30 Gy Involved-Field Radiation Therapy (IFRT), is suggested as the current standard of care. With most HL patients achieving a long lasting complete remission and long-term survival after therapy, the risk of long term complications, particularly second malignancies, such as breast and lung cancer [1,2] and cardiovascular disease [3,4] is of paramount importance.

Radiotherapy has substantially changed over the last 20 years in terms of radiation volumes and doses reduction. Two major critical points are currently under investigation: a further shrinkage of treatment volumes towards the involved-node(s) concept (INRT) [5] and the employment of highly conformal techniques such as Intensity-Modulated Radiation Therapy (IMRT). INRT can theoretically reduce radiation-induced late effects by reducing treatment volumes, while IMRT has the potential to substantially improve dose distribution, reducing the amount of healthy tissues exposed to intermediate-high dose level. Such a dosimetric gain could be particularly important in mediastinal disease [6], for which there is growing evidence that highly conformal irradiation modalities may improve critical organs sparing with clinically significant consequences.

We herein report on a comparative planning study of INRT actually delivered with 3D-CRT and different known IMRT solutions (HT and VMAT) in a cohort of young female patients affected with early stage HL and mediastinal involvement. The present study also introduces TomoDirect as a rapid, low-modulation solution of the Tomotherapy HI-ART II platform, and a specific volumetric IMRT approach designed in our Institution for lymphoma patients and named Butterfly-VMAT (B-VMAT, so called for typical butterfly-shaped low isodoses), based on multiple arcs (two coplanar arcs and one non-coplanar arc). The primary aim of the study was to investigate the most appropriate planning solution, in terms of plan quality (target coverage and OAR sparing) and treatment efficiency.

## Methods

Ten consecutive female patients affected with early stage HL with mediastinal involvement, submitted to radiotherapy from April 2008 to September 2009 at our Institutions, were enrolled onto the present study. Median age was 26.5 years old. All patients received standard combined modality treatment with 3 or 4 cycles of ABVD chemotherapy (respectively favourable or unfavourable early stages according to EORTC prognostic risk factors), followed by Involved Nodal Radiotherapy (INRT) at a dose of 30 Gy/15 fractions (according to EORTC H10 protocol radiotherapy guidelines). Two out of 10 patients had mediastinal bulky disease at presentation, 2/10 hilar involvement, 4/10 supraclavicular involvement, 6/10 supraclavicular and upper neck involvement. None of the patients had extranodal disease. Patients with axillary involvement were excluded.

All patients had pre and post-chemotherapy contrast-enhanced Computed Tomography (CT) and FDG-PET Computed Tomography (FDG-PET-CT) scans. All patients were contoured on Oncentra Masterplan TPS station Version 4.1 (Nucletron, Veenendal, The Netherlands). CT simulation scans were all non contrast-enhanced, with a slice thickness of 3 mm; images were acquired in treatment position, using immobilization devices usually consisting of thermoplastic masks.

Clinical Target Volume (CTV) and Organs-at-Risk (OAR) were contoured by the same 2 radiation oncologists.

INRT CTV was defined according to EORTC-GELA guidelines [1], as the pre-chemotherapy CTV (contoured on the basis of pre-chemotherapy CT and PET-CT scans) modified according to post-chemotherapy anatomic boundaries. As recommended by EORTC-GELA guidelines, major blood vessels and heart were excluded whenever possible. A 8 mm isotropic margin was added to CTV to generate Planning Target Volume (5 mm to compensate for target motion of mediastinal lymph nodes and 3 mm for set-up error, with a daily cone-beam CT IGRT protocol).

Lungs, thyroid, breasts, heart and coronary ostia were defined as organs at risk (OAR) and delineated on CT datasets; for the breast glandular tissue we used a standard window (0) and width (500) level. The heart was defined from the auricles to the tip of the organ, including thus all cardiac chambers. The origin of the coronary arteries was defined as the outer circumference of the proximal aorta, extending from the tip of the auricles to the bulb of the aorta (usually 2.5-3 cm, with slight individual differences). Non-target tissue was defined as the patient’s volume covered by the CT scan (external patient contour) minus the PTV.

Prescription dose was 30 Gy in 2 Gy daily fractions as mean PTV dose for all plans. As goal for dose homogeneity we intentionally decided not to follow recommendation of ICRU No. 83 in terms of dose coverage. The classical objectives defined by ICRU No. 50 (in which the target must be covered by more than 95% and less than 107% of the prescription dose), have been considered more appropriate in this setting (volumes close to skin surface, high radiosensivity, microscopic disease). The maximum tolerated dose was 115% of the prescribed dose (i.e., 34.5 Gy) in one voxel for the case of 3D-CRT; dose was reported to medium for all treatment modalities. Dose objectives for OAR (Table 1) were derived from a previously published study with the aim of a potential comparison [7]. Beam energy for all plans was 6 MV. All ten patients were actually treated with 3D-CRT and all treatments were then re-planned; therefore, five radiotherapy treatment plans were generated for each patient: 3D-conformal (3D-CRT) plan, single-arc VMAT plan (VMAT), three arcs VMAT plan (named “butterfly” VMAT, or B-VMAT), Helical Tomotherapy plan (HT) and TomoDirectplan (TD).

**Table 1:** Dose objectives for traditional dose optimization on HT and TD

**Structure**	**Parameter**	**Objective**
PTV	D_mean_(Gy)	30
	V_90%_ (%)	99
	V_95%_ (%)	95
	V_107%_ (%)	1
Breast	V_4Gy_ (%)	50
	V_10Gy_ (%)	33
Lung	V_5Gy_ (%)	50
	V_10Gy_ (%)	33
Thyroid	V_18Gy_ (%)	50
	V_25Gy_ (%)	33
Heart	V_7.7Gy_ (%)	50
	V_15Gy_ (%)	33
Coronary Ostia	V_20Gy_ (%)	100

Conventional 3D-CRT plans consisted of 2 anterior-posterior parallel opposed fields (gantry angles 0°-180°), shaped with multi-leaf collimators on beam’s-eye-view. Calculations were performed with Oncentra Masterplan using Collapsed Cone algorithm.

**Figure 1:**
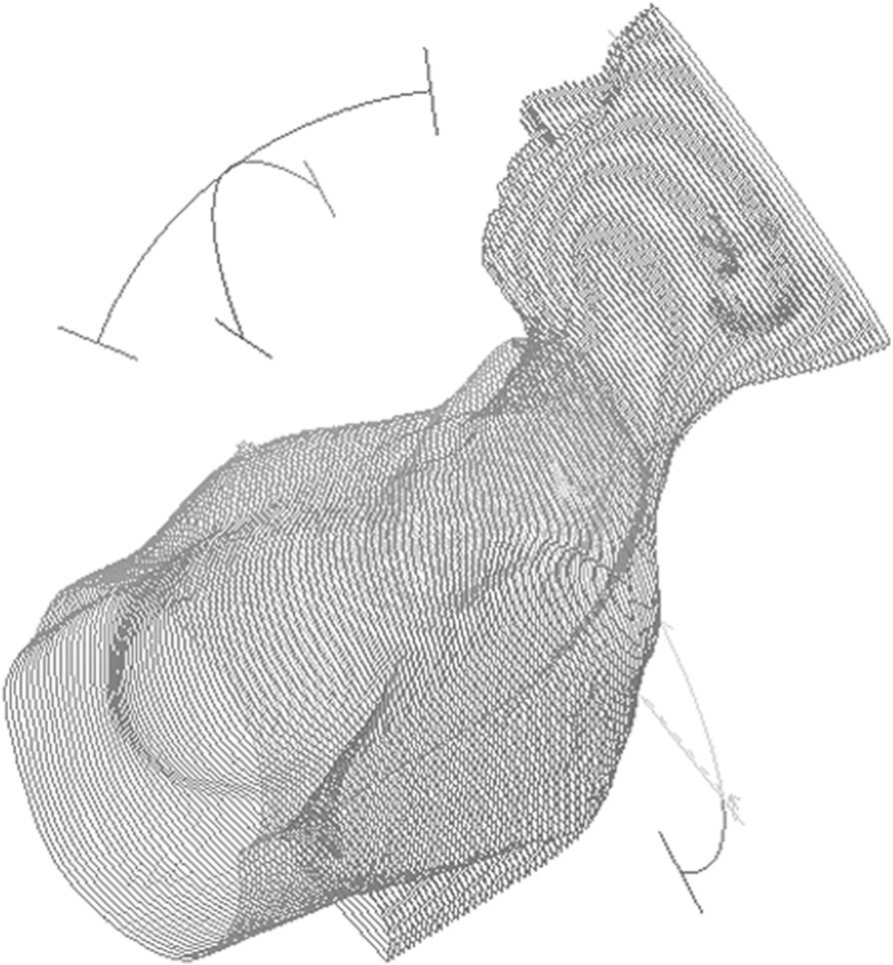
3D-graphical representation of the arc /beams configuration employed in Butterfly VMAT (B-VMAT) approach.

0a single arc of 360° (gantry starting angle 180°) or a 3 arcs plan (B-VMAT), developed and chosen in our Institution as preferred class solution in lymphoma patients with mediastinal involvement in order to reduce low doses radiation exposure (“bath doses”) to lungs and breasts; this approach consists of 2 coplanar arcs of 60° (gantry starting angles 150° and 330°) and 1 no-coplanar arc of 60° (gantry starting angles 330°, couch angle 90°) (Figure 1).

VMAT plans (both single-arc VMAT and B-VMAT) were computed on Elekta Monaco treatment planning system (TPS version 3.0). Monaco TPS allows a peculiar dose optimization by biological cost-functions for both PTV and OARs, using 3 main functions: the Poisson statistics cell-kill model, the serial complication model and the parallel complication model. Three-dimensional dose distributions are converted to either a EUD for the Poisson cell kill model and Serial complication model or a fraction of organ damaged for the parallel complication model [8]. These biological parameters are then included in the overall objective score for plan optimization. Each parameter is changed during optimization process to minimize the objective function describing coverage of PTV and sparing of each OAR. All VMAT plans were planned for Elekta Axesse Linear Accelerator, with its specific Beam Modulator (with 4 mm leaf width at isocenter) employed to achieve the desired beam’s fluence. The XVMC/VEF Monte Carlo algorithm with a 3% variance was used for all cases [9]. Calculation grid was set to 3 mm.

HT and TD plans were created and optimized using TomoTherapy Hi-Art version 4.0.5 TPS (Accuray, Inc., Sunnyvale, CA). For each plan, the treatment field width, pitch (the TD pitch is defined as the distance of couch travel in centimetres per sinogram projection) and modulation factor need to be selected. Then, the dose distribution for each beamlet that passes through the target is calculated by a convolution/superposition algorithm. Once the beamlet calculation step is completed, the optimization process begins and an iterative least-squares minimization method is used to optimize the objective function. During the final dose computation the optimized sinogram is converted to the delivery sinogram, taking into account for leaf fluence output factors and latency data. A fine calculation grid (256x256 pixels) was used both in the optimization and calculation processes.

HT treatments were planned with a modulation factor between 2.6 and 3.7, a pitch of 0.287 and a field width of 25 mm, that can be considered a suitable compromise between cranio-caudal dose spread (lower than a 50 mm field width) and treatment time (lower than a 10 mm field width).

In TD plans, only a two opposed beam configuration was investigated because it better reproduces the standard 3D-CRT technique. A planning modulation factor between 2.0 and 2.2 and a jaw width of 25 mm were used. The pitch value was set to the default value that is one tenth of the field width (2.5 mm/projection for the 25 mm beam).

Quantitative evaluation was performed by means of cumulative dose-volume histograms (DVHs). Analyzed parameters for PTV included D_mean_, V_95_, V_107_, Conformation Number (CN) and Homogeneity Index (HI). Plan conformity was evaluated using the CN proposed by van’t Riet et al. [10] which takes into account the overlap of the prescription isodose line with the PTV as well as the dose spilling over normal tissue. CN is defined as *CN = TV*^*2*^_*PI*_*/ TV*^*x*^*V*_*PI*_ where V_PI_ is the volume of the prescription isodose, TV is the planning target volume, and TV_PI_ is the planning target volume covered by the prescription isodose. The maximum CN value is 1 as it is achieved for an ideal plan [11]. Homogeneity index was defined by the equation *HI =(D*_*2*_*-D*_*98*_*)/D*_*p*_ where D_2_ and D_98_ are respectively doses received by 2% and 98% of volume while D_p_ is the prescription isodose.

Analyzed parameters for OAR were mean dose and representative V_d_. For each plan the delivery treatment time was estimated. The Wilcoxon matched-paired signed-rank test was used to compare the results among all different 5 techniques. The threshold for statistical significance was p≤0.05. All statistical tests were two-sided and were performed using the Systat version 7.0.1 (Systat Software, Inc.225 Chicago, USA).V_4_, V_5_ and V_20_ for lungs and breasts were plotted with CN by scatterplot.

## Results

**Figure 2:**
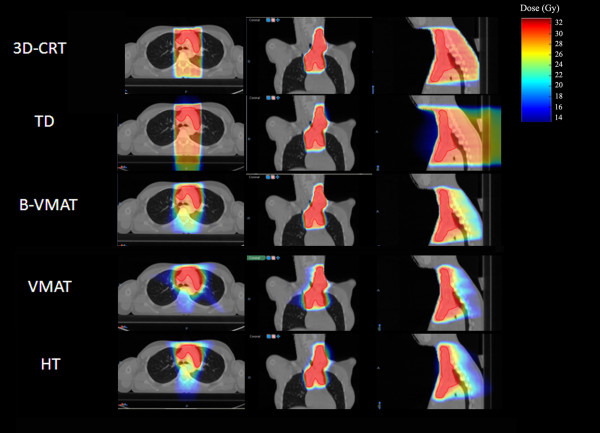
Dose distributions achievable in patient number 4 with all different five techniques.

Figure 2 represents the dose distributions in a single patient with all different five techniques while detailed results of the planning comparison for the 5 different techniques in all 10 patients (mean values) are shown in Table 2.

**Table 2:** Comparison of mean dosimetric parameters computed for 3D-CRT, TD, B-VMAT, VMAT and HT

**Variable**	**Objectives**	**3D-CRT**	**TD**	**B-VMAT**	**VMAT**	**HT**
**PTV**						
Volume (cm^3^)	428.7 ± 195.6					
D_Mean_	30	30.6±0.7^b,e^	30.0±0.1^a,c,d^	30.4±0.2^b,e^	30.4±0.2^b,e^	29.9±0.1^a,c,d^
V_90% (%)_	99	98.2±1.0^b,d,e^	99.2±0.7^a,c,d^	98.3±1.2^b,e^	98.6±1.1^b,e^	99.1±0.2^a,c,d^
V_95% (%)_	95	94.8±0.5^b,d,e^	97.4±1.7^a,c,d,e^	95.5±2.3^b^	95.4±1.7^b^	95.9±0.7^a,b^
V_107% (%)_	<1	5.0±5.2^b,d,e^	0.02±0.03^a,c,d,e^	5.5±3.8^b,e^	5.50±3.80^b,e^	0.3±0.28^a,b,c,d^
CN_95% (%)_	1	0.30±0.1^c,d,e^	0.30±0.1^c,d,e^	0.67±0.1^a,b,d,e^	0.76±0.02^a,b,c^	0.77±0.06^a,b,c^
HI	0	0.30±0.1^b,c,d,e^	0.10±0.03^a,c,d,e^	0.20±0.1^a,b,e^	0.16±0.04^a,b,e^	0.07±0.01^a,b,c,d^
**Lung**						
D_mean(Gy)_	-	6.6±2.6	6.3±2.7	5.9±2.5	6.4±2.5	5.9±2.2
V_5Gy (%)_	50	29.3±12.4^b,c,d,e^	26.4±11.6^a,c,d,e^	31.3±14.3^a,b,d,e^	39.0±15.6^a,b,c^	37.0±12.6^a,b,c^
V_10Gy (%)_	33	22.6±9.4^d^	21.9±9.6^d^	20.8±10.0^d^	25.6±13.3^a,b,c,e^	20.6±11.0^d^
V_15Gy (%)_	-	18.6±7.6^c,d,e^	18.5±8.2^c,d,e^	15.3±8.0^a,b,e^	15.0±10.6^a,b,e^	12.8±7.5^a,b,c,d^
V_20Gy (%)_	-	15.2±6.2^c,d,e^	15.6±6.9^c,d,e^	11.3±6.7^a,b,d,e^	8.8±6.3^a,b,c,^	7.6±4.5^a,b,c^
V_30Gy (%)_	-	3.5±2.7^b,c,d,e^	0.7±0.5^a^	0.8±0.8^a^	0.3±0.3^a^	0.1±0.3^a^
**Breast**						
D_mean(Gy)_	-	1.0±0.4^c^	0.9±0.9^c^	0.7±0.3^a,b,d,e^	0.9±0.4^c^	1.2±0.9 ^c^
V_4Gy (%)_	50	4.5±2.3^d,e^	3.7±4.2^d,e^	4.6±3.0^d,e^	6.2 ±4.7^a,b,c^	5.4±5.5^a,b,c^
V_10Gy (%)_	33	2.5±1.4^c,d,e^	2.4±3.0^c,d,e^	1.1±1.0^a,b^	1.2±1.1^a,b^	1.4±1.8^a,b^
V_15Gy (%)_	-	1.4±0.7^c,d,e^	1.6±2.2^c,d,e^	0.4±0.4^a,b^	0.3±0.4^a,b^	0.6±1.0^a,b^
V_20Gy (%)_	-	0.9±0.5^c,d^	1.3±1.9^c,d^	0.1±0.1^a,b^	0.1±0.1^a,b^	0.2±0.5^a,b^
V_30Gy (%)_	-	0.2±0.3^c,d,e^	0.6±1.0^c,d,e^	0^a,b^	0^a,b^	0^a,b^
**Thyroid**						
D_mean(Gy)_	-	17.7±4.5^c,d,e^	17.3±5.1^c,d,e^	13.5±6.3^a,b^	13.5±5.3^a,b^	14.4±5.5^a,b^
V_18Gy (%)_	50	45.9±18.1^c,d,e^	51.3±19.2^c,d,e^	35.2±21.6^a,b^	33.4±18.8^a,b^	38.2±23.0^a,b^
V_25Gy (%)_	33	32.0±14.4^c,d,e^	32.9±14.6^c,d,e^	22.2±15.8^a,b^	21.3±14.7^a,b^	24.5±19.8^a,b^
**Coronary Ostia**						
D_mean(Gy)_	-	22.1±9.9^c,d,e^	19.8±9.3^c,d,e^	15.7±7.9^a,b^	15.9±8.9^a,b^	16.4±8.4^a,b^
V_20Gy (%)_	100	73.5±40.8^c,d,e^	59.1±37.7^c,d,e^	43.1±32.6^a,b^	44.0±34.3^a,b^	44.3±34.2^a,b^
**Heart**						
D_mean(Gy)_	-	5.1±4.0^c,d,e^	4.3±3.7^c,d,e^	3.5±2.3^a,b^	3.1±2.2^a,b^	3.8±3.8^a,b^
**Non target tissue**						
D_mean(Gy)_	-	4.1±1.3	3.8±0.9	3.1±0.7	3.6±0.9	3.90±0.96
**Time (min)**	-	3.2±1.1^b,c,d,e^	4.4±1.1^a,c,d,e^	12.0±2.8^a,b,d,e^	6.2±1.3^a,b,c,e^	7.2±1.6^a,b,c,d^

Statistically significant differences (p<0.05) for each technique versus alternatives are reported: ^a^Test vs 3D-CRT; ^b^ Test vs TD; ^c^ Test vs B-VMAT; ^d^ Test vs VMAT; ^e^ Test vs HT.

### PTV

All dose and volumes data were calculated as mean values of the 10 considered patients. Mean doses to the PTV were almost identical. D_mean_ varied from a minimum value of 29.9 Gy for HT to a maximum value of 30.6 Gy for 3D-CRT. The best target coverage according to ICRU 50 definitions was obtained with HT and TD (V_90%_ = 99.1 and 99.2, respectively), even if the target coverage was optimal also for 3D-CRT, VMAT and B-VMAT, as shown in Table 2. 3D-CRT, B-VMAT and VMAT techniques had higher values of V_107%_ (5%, 5.5% and 4.3%). Conformation Number reached a maximum value of 0.77 with HT, followed by VMAT with 0.76; B-VMAT had an intermediate value of 0.67, while TD achieved CN value of 0.3 like 3D-CRT. Homogeneity Index values of HT (HI = 0.07) and TD (HI = 0.1) were lower than all other techniques.

### Lungs

**Figure 3:**
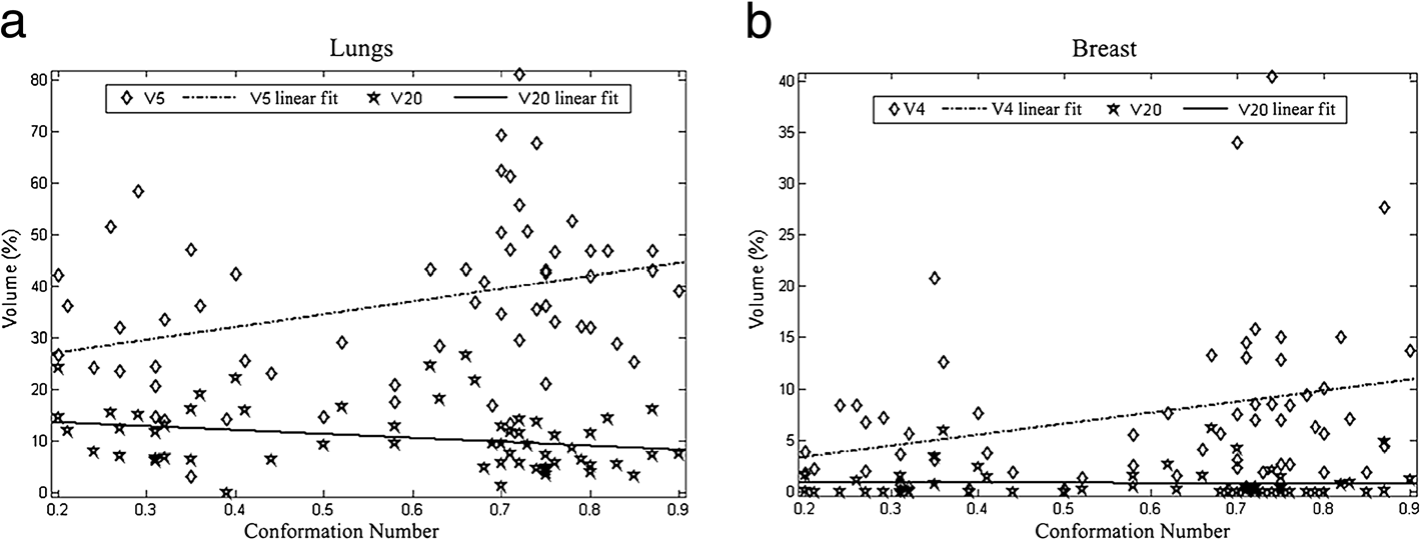
Lung and breast volumes (%) receiving 4–5 Gy and 20 Gy plotted as a function of CN for all available different techniques.

D_mean_ showed similar values for all different techniques, including 3D-CRT. For low doses (V_5_), a trend of increased volumes may be observed from 3D-CRT (mean value of 29.3%) to VMAT (mean values of 39.0%). For volumes receiving higher doses (V_20_), the maximum value was reached by TD (V_20_ = 15.6 %), with lowest values for IMRT techniques with the highest CN. Figure 3 shows the correlation between CN values and V_5_ - V_20_values: a trend for a correlation with CN is appreciable.

### Breasts

D_mean_ showed comparable values between HT and VMAT (1.2 ± 0.9 and 0.9 ± 0.4), but significant difference in terms of reduced mean dose of B-VMAT (p<0.05) compared with all others techniques may be observed (Table 2).

V_4_ values for 3D-CRT and TD were respectively 4.5% and 3.7%, while for VMAT and HT V_4_ values were respectively 6.2% and 5.4%. For B-VMAT, the V_4_ value was similar to 3D-CRT (4.6%). V_15_ and V_20_were low for all different techniques (range 0.3-1.6 for V_15_ and 0.1-1.3 for V_20_). Figure 3 shows the correlation between CN values and V_4_ and V_20_; a trend for an inverse correlation is appreciable only for V_4_.

### Thyroid gland

D_mean_ for thyroid gland were lower for HT, VMAT and B-VMAT (14.4, 13.5 and 13.5 Gy) compared to TD and 3D-CRT (17.3 and 17.7 Gy). From the analysis of the values of each single patient (not reported in the manuscript), come out as only techniques able to achieve higher CN values (more than 0.6) are ever able to satisfy the dose objectives indicated for thyroid.

### Coronary Ostia

Mean dose as well as V_20_ values were comparable for all techniques; treatment plans with higher CN values did not translate into a substantial sparing of this organ at risk, as in most of cases coronary ostia were distant from target volumes.

### Heart

Mean Dose received by the whole heart was similar (range 3.1 – 5.1 Gy) for all different techniques.

### Non target tissue

No significative differences were found in terms of D_mean_ to the whole considered body; however the minimum value was reached by B-VMAT technique (3.1 Gy) while 3D-CRT had the maximum mean value of 4.1 Gy.

### Treatment efficiency

All techniques had clinically acceptable treatment times. IMRT techniques showed higher treatment times (a mean values of 7.2 minutes for HT, 6.2 minutes for VMAT (single arc) and 12 minutes for B-VMAT), while 3D-CRT and TD were significantly faster (3.2 and 4.4 minutes).

#### Conformation number

Each planning method gives different values of CN based on its degree of modulation. Figures 3a and 3b show the trend of “low” doses (V_4_,V_5_) and “high” doses (V_20_) as a function of CN for all different techniques, respectively for lungs (Figure 3a) and breast (Figure 3b). For lungs, volumes receiving low doses (V_5_) started from 20% for lower CN techniques (corresponding to 3D-CRT and TD) to 50% values for higher CN techniques. An opposite trend is shown for volumes receiving “high” doses (V_20_), shifting from 15% for lower CN approaches to roughly 5% for higher CN techniques (HT and VMAT). For breast tissues, a same trend was evident for volumes receiving low doses (V_4_), but not for volumes receiving higher doses (V_20_).

## Discussion

There is growing evidence that highly conformal irradiation modalities, like different IMRT approaches, may improve critical organs sparing by improving conformity [6,7,12-15], with potentially clinical significant consequences in haematological malignancies.

The open question is whether IMRT might lead to a lower dose to the surrounding organs and whether the rate of late complications (in particular lung and cardiac toxicity) could be really reduced with IMRT.

In this comparative planning study, we evaluated different photons IMRT solutions applying the INRT concept, in comparison with the actual reference technique 3D-CRT.

Helical Tomotherapy and VMAT were tested as very highly conformal techniques for PTV coverage, with also the potential advantage of a faster delivery solution for VMAT (one rotation only). B-VMAT was specifically developed in our Institution for HL patients with mediastinal involvement, in order to achieve a highly conformal solution lowering radiation exposure of lungs and breasts. TD represented a feasible and simple alternative planning solution for Tomotherapy if a low degree of modulation is desired.

All different IMRT techniques were able to better conform the dose to PTV compared to 3D-CRT, even if the standard 3D-CRT approach lead to a valid target coverage, satisfying ICRU criteria (V_95%_> 95%); better PTV coverage achieved by IMRT is depending of course on the IMRT ability, regardless of different technical approaches, to modulate the intensity of each radiation beam, resulting on a higher conformal delivery of radiation dose to PTV. Among different IMRT techniques, HT and VMAT showed the best level of conformity to PTV (Table 2, p<0.05 for CN); VMAT and B-VMAT had higher values of the maximum dose (V_107%_), however better results in terms of homogeneity of dose inside PTV are promised for future version of Monaco software. All different IMRT solutions were also better in terms of lowering mean doses to certain OAR (thyroid gland, heart and coronary ostia,), as already shown by other Authors in similar planning comparison studies[7,12,13]. For such organs, in which probably a lower mean dose is potentially correlated to a lower incidence of late toxicity, IMRT confirms its advantage in our series, with all different modulated techniques able to better spare thyroid gland; it is reasonable that lowering the radiation dose to the thyroid gland could reduce the risk of late toxicity as hypothyroidism and second cancer [16].

A substantial avoidance of heart and coronary ostia has been however achieved independently from IMRT, by simply applying the INRT concept other than the wider IFRT approach (in 8 out of our 10 patients, these structures received very low doses), as previously shown by Koeck et al. [17], reporting no significant difference in cardiac D_mean_ between 3D-CRT and IMRT when using INRT. Some authors stated that the aim of heart sparing could be best achieved with IMRT [18]. Anyway, the individual magnitude of clinical benefit related to a possible IMRT dosimetric gain is hard to predict, mainly depending on adjunctive risk factors (patient anatomy, entity of mediastinal involvement, cardiac comorbidity). In the near future, at least in critical anatomical presentations and in clinical cases at risk for cardiac toxicity, new different approaches, such as proton therapy, could probably further reduce cardiac radiation doses (a strong reduction of heart exposure as well as of radiation doses to critical cardiac subunits was reported by Hoppe et al. [19]).

One of the aims of IMRT in supradiaphragmatic HL patients is to reduce radiation exposure to lungs; D_mean_ to lungs was similar for all different technical approaches, including 3D-CRT; all IMRT techniques were able to significantly reduce volumes receiving high doses (>20 Gy); this could be expected to translate into a lower incidence of acute radiation pneumonitis (Girinsky et al. [12] reported a grade >2 lung toxicity in 10% of the cases with mean dose of 12.8 Gy and in 5% with average V_20_ of 25% in treating HL patients with mediastinal masses). Opposite to a reduction of V_20_ values, IMRT techniques resulted in an increase of V_5_ parameter. For low doses pulmonary volumes (V_5_), higher was the CN of each different technique, higher were the corresponding V_5_ values; on the other hand, there was an inverse correlation between CN and lungs V_20_ (as the CN decreases, higher V_20_ values are obtained). The slope of the correlation curves shown in Figure 3 suggests that the CN achievable with different techniques could be considered as a potential endpoint (the best compromise between low and high doses volumes) for planning.

The impact of low dose radiation exposure to large breasts’ volume on the risk of developing a secondary cancer is currently unknown; there are some clear data showing a risk reduction with lower doses and smaller volumes [20], but there are still uncertainties on which dose distribution to the breasts is the most conservative [21]. Comparing D_mean_ between 3D-CRT and IMRT we find comparable results; in our experience the B-VMAT solution, specifically designed as our class solution for lymphoma patients involving the mediastinum, seems to be the most appropriate, obtaining D_mean_ values of 0.7 Gy (little bit lower than those achieved with other planning solutions, p<0.05). D_mean_ values obtained in our study with all different techniques are in any case comparable to those reported in other experiences [21], even if with a broad range of V_4_ and CN values, strongly dependent on patients’ specific anatomy (Figure 3).

The dose to non-target tissue did not significantly vary among all different techniques (slightly higher for techniques with higher CN values).

Although some experience are reported in literature [22] about the comparison of treatment plans resulting from optimization methods based on physical dose or biological parameters, the present study is the first experience evaluating volumetric IMRT techniques based on radiobiological planning in treating HL patients, as well as the first experience comparing them with Tomotherapy-based solution (classical HT); radiobiological optimization, specifically part of the Monaco IMRT planning process, was able to respect all dose constraints and to obtain satisfactory dose distribution, both for PTV coverage and OAR’s sparing. A planning option that has not been investigated in the present study, but that can achieve similar dose distribution, is traditional IMRT with equidistant fields, an approach already tested by other groups with the use of 9 static coplanar IMRT fields [7].

IMRT reduces high doses to OAR to a varying degree, depending on prescription strategies and target paradigm, at the cost of larger volumes irradiated to low/intermediate doses, whose clinical significance is nowadays unknown. Considering all different OAR’s together, we were not able to find out an optimal IMRT technique, with constantly better dosimetric performances; one of the reasons could stand in the anatomical differences between 10 included patients, as well as in the INRT approach itself, able to achieve a good sparing of most OAR even with less conformal techniques [23]. In this setting, IMRT represents a continuum of possible dose distributions, being the choice of the specific technique apparently of minor importance. In our clinical routine the decision to use IMRT or not is made on an individual basis after comparative treatment planning, with the largest benefit to be expected in patients with large mediastinal targets.

A future generation of studies would probably consider different IMRT solutions for different disease presentations at diagnosis, including second cancer risk modeling in the planning process.

## Conclusions

IMRT techniques showed superior target coverage and OAR sparing compared to 3D-CRT, with, as expected, larger volumes of some healthy tissues (especially lungs and breasts) receiving low-intermediate doses.

Among different IMRT techniques, HT and VMAT showed the highest levels of conformity; TD performances were, as expected, very similar to those of 3D-CRT; B-VMAT and HT emerged as the planning solutions able to obtain the most appropriate compromise and balance between conformity around the target (typical of IMRT) and limited volumes of OAR receiving low-intermediate radiation doses (typical of 3D-CRT).

## Competing interests

The authors declare that they have no competing interests.

## Authors’ contributions

CF, ARF, PC, AB, PCM, PF VCB and UR contributed in the production of data and in the manuscript writing. CF and RR participated in the collection, assembly and analysis of data. ST and UR gave final revision and approval. All authors read and approved the final manuscript.
